# Prognostic and clinicopathological significance of systemic immune-inflammation index in upper tract urothelial carcinoma: a meta-analysis of 3911 patients

**DOI:** 10.3389/fonc.2024.1342996

**Published:** 2024-06-14

**Authors:** Ziyi Yu, Zhencheng Xiong, Jinchao Ma, Peng Du, Shuo Wang, Jia Liu, Yudong Cao, Yong Yang

**Affiliations:** ^1^ Key Laboratory of Carcinogenesis and Translational Research (Ministry of Education), Urological Department, Peking University Cancer Hospital and Institute, Beijing, China; ^2^ Trauma Medical Center, Department of Orthopedics Surgery, West China Hospital, West China Medical School, Sichuan University, Chengdu, China

**Keywords:** upper tract urothelial carcinoma, systemic immune-inflammation index, prognosis, biomarker, meta-analysis

## Abstract

**Background:**

Systemic immune-inflammation index (SII), a novel prognostic indicator, is being more commonly utilized in different types of cancer. This research project involved combining information from previously published studies to examine how pre-treatment SII can predict outcomes in individuals with upper tract urothelial carcinoma (UTUC). Further examination of the correlation between SII and clinical and pathological features in UTUC.

**Methods:**

We thoroughly chose pertinent articles from various databases including PubMed, Embase, Cochrane Library, Web of Science, Chinese National Knowledge Infrastructure (CNKI), WanFang database, and Chinese Scientific Journal Database (VIP) until March 10, 2022.The data collected was analyzed using Stata 17.0 software (Stat Corp, College Station, TX). Subsequently, the impact of SII on the survival outcomes of UTUC patients was evaluated by combining HRs with 95% confidence intervals.

**Results:**

Six included studies were finally confirmed, including 3911 UTUC patients in seven cohorts. The results showed that high SII before treatment predicted poor overall survival (HR =1.87, 95%CI 1.20-2.92, p=0.005), cancer specific survival (HR=2.70, 95%CI 1.47-4.96, P=0.001), and recurrence‐free survival (HR =1.52, 95%CI 1.12-2.07, P=0.007). And the elevated SII may be related to LVI (present *vs*. absent) (OR=0.83, 95% CI=0.71-0.97, p=0.018), pT stage (pT ≥3 *vs*. < 3) (OR=1.82, 95% CI=1.21-2.72, p=0.004), and pN stage (N+ *vs*. N0) (OR=3.27, 95% CI=1.60-6.71, p=0.001).

**Conclusion:**

A comprehensive analysis of all included articles in this study showed that higher pretreatment SII was related to poorer survival outcomes and adverse pathological features independently.

**Systematic review registration:**

https://www.crd.york.ac.uk/prospero/, identifier CRD42022316333.

## Introduction

Upper tract urothelial carcinoma (UTUC) is a rare and highly heterogeneous disease which accounts for approximately 5% of all urothelial malignancies (1, 2). In China, the figure may be even higher, accounting for 17.9% of urothelial cancers (3). The incidence of this disease varies in different regions. UTUC is more likely to invade surrounding tissues and metastasize due to the thin ureteral wall and abundant periductal lymph. Because of this, it has a poor prognosis and often has more aggressive biological characteristics, and its five-year survival rate is about only 60% (4). Radical nephroureterectomy (RNU) has been the main treatment option because of the absence of standardized clinical diagnosis and treatment guidelines. Despite timely completion of standard radical surgery, the outlook remains grim, as 22% to 47% of patients experience intravesical recurrence postoperatively (5). Thus, it is necessary to investigate biomarkers that can be monitored after RNU.

An increasing amount of studies have concentrated on the involvement of the immune system in every stage of cancer progression (6). Inflammation may lead to alterations in the levels of different cells associated with inflammation in the body, including leukocytes, erythrocytes, and thrombocytes. Blood markers of inflammation like platelet-lymphocyte ratio (PLR), neutrophil-lymphocyte ratio (NLR), and lymphocyte-monocyte ratio (LMR) have been utilized for assessing inflammation levels in the body and predicting the likelihood of recurrence and prognosis in urinary system cancers (7–10). The systemic immune-inflammation Index (SII), a novel inflammatory marker, has gained increasing recognition and acceptance in recent times. It is determined by the formula SII = platelet count × neutrophil count/lymphocyte count. It has more excellent clinical value than previous inflammatory indicators because it simultaneously contains three kinds of peripheral blood cells. To date, there have been no published meta-analyses examining the effects of SII on the prognosis of UTUC. Hence, the objective of this research was to methodically examine the predictive importance of SII in individuals diagnosed with this condition.

## Materials and methods

### Protocol and ethics

The research was carried out following the guidelines of the Preferred Reporting Items for Systematic Reviews and Meta-Analyses (PRISMA) (11) and was registered in PROSPERO (registration number CRD42022316333) in advance. All data in this study were secondary analyses of previous studies and therefore did not require ethical approval and signed patient informed consent.

### Search strategy

Two researchers (Z.Y. and Z.X.) conducted an extensive search of various databases including PubMed, Cochrane Library, Embase, Web of Science, Chinese National Knowledge Infrastructure (CNKI), WanFang database, and Chinese Scientific Journal Database (VIP) until March 10, 2022 independently, and discuss any differences with a third researcher (J.M.). This meta-analysis utilized the search terms (upper tract OR ureter OR ureteral OR renal pelvis OR renal pelvic OR ureteral neoplasms OR urothelium) AND (systemic immune-inflammation index OR SII). We carefully screened and reviewed the literature by title and abstract, and initially eliminated the nonconforming literature. Then we further screened the selected literature by reading the full text. Furthermore, we conducted a manual search of the reference lists of pertinent studies in hopes of identifying additional suitable articles.

### Criteria for inclusion and exclusion

All studies had to meet specified criteria to be included in this meta-analysis, as follows: (i) The studies examined how the pretreatment systemic immune-inflammation index (SII) correlates with the prognosis of patients with upper tract urothelial carcinoma confirmed through histological analysis. (ii) The complete text of the articles included specified the exact threshold value for SII; (iii) Survival outcomes such as overall survival (OS), cancer-specific survival (CSS), or recurrence-free survival (RFS) for patients with UTUC are assessed for availability. (iv) Patient prognosis hazard ratio (HR) and 95% confidence interval (CI); (v) English or Chinese full‐text articles.

Publications will be excluded if they refer to the following situations: (i) Poster sessions, reviews, letters, case reports, conference abstracts, or comments; (ii) Incomplete or unavailable data; (iii) Animal experiments or basic research; (iv) Duplicate articles.

### Extracting and analyzing data

From the included literature that met the criteria, the following information was extracted: author’s name, publication year, the region of study population, study design, study period, sample size, patient age, follow-up time, cut-off value, clinical stage, prognostic indicators (OS\CSS\RFS), and HRs with the corresponding 95% CIs.

In this meta-analysis, pooled HRs with 95% confidence intervals were assessed by two methods:(a) Extracting HRs with 95% CIs directly from articles through univariate and multivariate analysis; (b) Multivariate analysis was favored for its higher accuracy in selecting HRs.

The quality of the study was assessed using the Newcastle-Ottawa scale (NOS) (12). The NOS score is on a scale of 0 to 9, and studies with a score of 6 or higher were considered high quality researches.

### Statistical analysis

The data collected was analyzed using Stata 17.0 software (Stat Corp, College Station, TX). The prognostic significance of SII on the survival of UTUC patients was assessed through pooled HRs and 95% CIs. SII was deemed a significant predictor if the pooled 95% confidence interval did not intersect with 1 and had a p-value less than 0.05. Pooled HRs>1 with 95% CIs, not including 1, means that patients with higher SII have a poorer prognosis. The study’s heterogeneity was assessed using Cochran’s Q-test and Higgin’s I² statistic. An I² value greater than 50% or a p-value less than 0.10 signified notable heterogeneity. A random-effect model was employed in this article. Furthermore, subgroup analysis and sensitivity analysis were employed to examine the possible sources of variation and evaluate the consistency of the findings. Publication bias was visually assessed using Begg’s and Egger’s tests. An examination was conducted to analyze the connection between SII and clinical and pathological characteristics by combining the odds ratios (ORs) and their corresponding 95% confidence intervals (CIs). Pooled ORs>1 with 95% CIs not including 1 means that higher SII is related to poorer pathological type. A P value less than 0.05 signifies the statistical importance of the findings in the study.

## Results

### Search results

**Figure 1:**
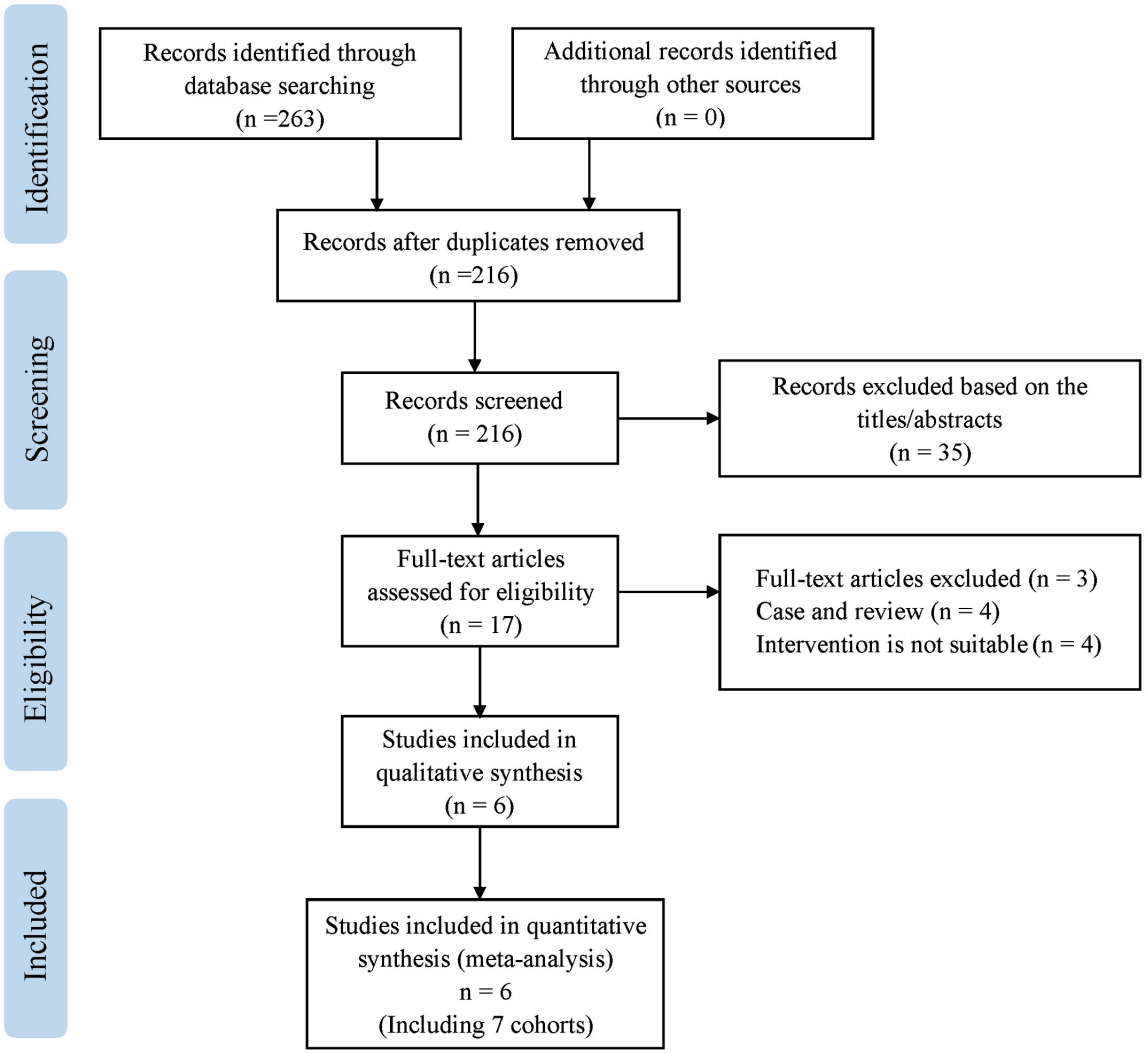
Meta-analysis flow diagram of included studies.

We initially retrieved 263 articles, and after eliminating duplicates, 216 articles remained. After reviewing the title and abstract, articles that did not meet the criteria were eliminated, leaving 17 articles to be read in their entirety. Six studies were finally confirmed, including 3911 UTUC patients in seven cohorts (13–18). **Figure 1** depicts the process of retrieving information.

### Characteristics of the studies included

The basic information description of the above-included literature is summarized in **Table 1**. All the articles included in the study were published within the past 5 years and were retrospective in nature. Two of the studies (14, 18) had three cohorts in China, two (13, 17) in Taiwan China, one (16) in Japan, and one (13) in Europe and the United States. Among the above six articles, there is one article in Chinese (18) and five in English (13–17). The total sample size for all studies was 3911, with included studies ranging from 103 to 2373. The average age of the patients was above 65 years, with thresholds varying from 410.3 to 672.44. Among them, five cohorts examined the correlation between OS and SII (13–16), while six cohorts analyzed the association between CSS and SII (13–17), and five cohorts investigated the link between RFS and SII (14, 15, 17, 18). Every study that was included in the analysis was deemed to be of excellent quality, scoring 6 or above on the Newcastle-Ottawa Scale (**Table 2**).

**Table 1:** Main characteristics of all included studies in the meta-analysis.

Author	Year	Region	Study design	Study period	Sample size	Age (years)	Follow-up time (months)	Cut-off value	Survival outcomes	Clinical stage	NOS
Jan (13)	2018	Taiwan	Retrospective	2007-2017	424	Median 70 (29-96)	Median 35 (IQR 14–60)	580	OS、CSS	T1-T4	8
Zheng (14)	2020	China	Retrospective	2006-2015	253 (TC)	Mean 67.6 ± 10.5	Median 33.8 (IQR 16.7-64.4)	672.44	OS、CSS、RFS	T1-T4	8
				2004-2016	272 (VC)	Mean 65.9 ± 10.3	Median 44.6 (IQR 26.8-65.3)	672.44	OS、CSS、RFS	T1-T4	8
Mori (15)	2020	USA/Europe	Retrospective	1990-2008	2373	Median 69	Median 38	485	OS、CSS、RFS	Ta-T4	9
Kobayashi (16)	2021	Japan	Retrospective	2004-2020	103	Median73 (IQR 68-78)	Median 41 (IQR 20–60)	520	OS、CSS	Ta-T4	7
Chien (17)	2021	Taiwan	Retrospective	2001-2013	376	Median 69 (IQR 15)	Median 52 (IQR 16.7)	485	CSS、RFS	Ta-T4	7
Zhang (18)	2021	China	Retrospective	2014-2020	110	Mean 66	Median 24	410.3	RFS	Ta-T4	6

TC, training cohort; VC, validation cohort; OS, overall survival; CSS, cancer-specific survival; RFS, recurrence-free survival.

**Table 2:** The Newcastle-Ottawa Scale (NOS) for assessing the quality of cohort studies.

Study	Selection	Comparability	Outcomes	Total Scores (Maximum 9)
Jan et al., 2018 (13)	3	2	3	8
Zheng et al., 2020 (14)	3	2	3	8
	3	2	3	8
Mori et al., 2020 (15)	4	2	3	9
Kobayashi et al., 2021 (16)	3	2	2	7
Chien et al., 2021 (17)	3	2	2	7
Zhang et al., 2021 (18)	2	2	2	6

### The impact of SII on OS in UTUC

The analysis included five groups consisting of 3425 patients to examine the correlation between preoperative SII and survival in UTUC patients (13–16).

The findings indicated that elevated systemic immune-inflammation index prior to therapy was associated with worse overall survival outcomes (HR =1.87, 95%CI: 1.20-2.92, p=0.005).

**Figure 2:**
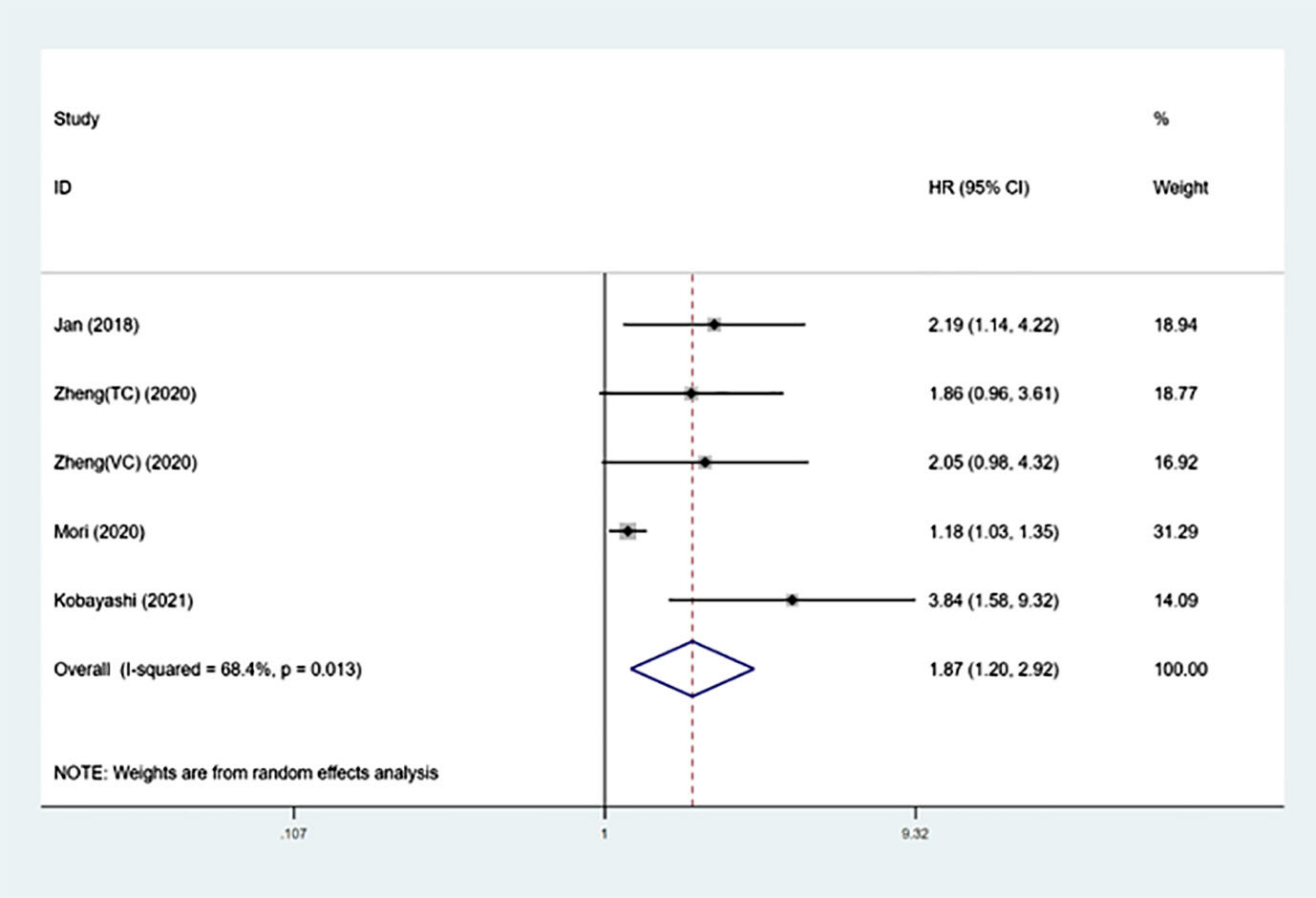
Forest plot of the association between SII and OS in patients with UTUC.

Because of the higher heterogeneity (I²= 68.4%, p=0.013), a random-effect model was performed (**Figure 2** and **Table 3**). After that, subgroup analysis was performed by region, sample size, cut-off value and follow-up time. **Table 3** shows that poorer OS was significantly associated with higher SII in subgroups with samples size >300 (p<0.001), cut-off value > 550 (p<0.001), and median follow-up time>40 months (p=0.001).

**Table 3:** Subgroup analysis of OS, CSS and RFS.

Outcome	Variables	No. of cohorts	No. of patients	Model	HR (95%CI)	p	Heterogeneity	
							I² (%)	p
OS								
Total		5	3425	Random	1.87 (1.20, 2.92)	0.005	68.4%	0.013
Region	Asia	4	1052	Random	2.26 (1.57, 3.24)	<0.001	0.0%	0.620
	USA & Europe	1	2373	/	1.18 (1.03, 1.35)	0.016	/	/
Sample size	>300	2	2797	Random	1.48 (0.82, 2.65)	0.191	69.8%	0.069
	<300	3	628	Random	2.28 (1.48, 3.52)	<0.001	0.0%	0.414
Cut-off value	>550	3	949	Random	2.03 (1.37, 3.01)	<0.001	0.0%	0.941
	<550	2	2476	Random	1.96 (0.62, 6.14)	0.251	85.0%	0.010
Follow-up time	>40 months	2	375	Random	2.68 (1.46, 4.90)	0.001	11.0%	0.289
	<40 months	3	3050	Random	1.53 (1.00, 2.34)	0.052	58.9%	0.088
CSS								
Total		6	3801	Random	2.70 (1.47, 4.96)	0.001	78.0%	<0.001
Region	Asia	5	1428	Random	3.32 (2.22, 4.95)	<0.001	0.0%	0.721
	USA & Europe	1	2373	/	1.21 (1.02, 1.43)	0.027	/	/
Sample size	>300	3	3173	Random	2.18 (0.96, 4.97)	0.064	79.3%	0.008
	<300	3	628	Random	3.29 (1.93, 5.60)	<0.001	3.4%	0.355
Cut-off value	>550	3	949	Random	2.91 (1.79, 4.72)	<0.001	0.0%	0.872
	<550	3	2852	Random	2.69 (0.91, 7.97)	0.074	84.3%	0.002
Follow-up time	>40 months	3	1127	Random	3.71 (2.14, 6.43)	<0.001	0.0%	0.480
	<40 months	3	3050	Random	1.99 (0.97, 4.08)	0.060	76.1%	0.015
RFS								
Total		5	3384	Random	1.52 (1.12, 2.07)	0.007	47.4%	0.107
Region	Asia	4	1011	Random	1.78 (1.30, 2.44)	<0.001	0.0%	0.506
	USA & Europe	1	2373	/	1.18 (1.01, 1.37)	0.033	/	/
Sample size	>300	2	2749	Random	1.32 (0.95, 1.84)	0.099	52.8%	0.145
	<300	3	635	Random	1.87 (1.18, 2.95)	0.008	11.8%	0.322
Cut-off value	>550	2	525	Random	1.58 (0.98, 2.53)	0.059	0.0%	0.915
	<550	3	2859	Random	1.62 (0.99, 2.64)	0.054	70.3%	0.034
Follow-up time	>40 months	2	648	Random	1.65 (1.12, 2.42)	0.011	0.0%	0.810
	<40 months	3	2736	Random	1.62 (0.93, 2.81)	0.088	64.3%	0.061
Language	English	4	3274	Random	1.27 (1.08, 1.49)	0.003	4.9%	0.369
	Chinese	1	110	/	3.65 (1.36, 9.80)	0.010	/	/

^OS, overall survival; CSS, cancer-specific survival; RFS, recurrence-free survival; HR, hazard ratio; CI, confidence interval.^

### Prognostic significance of SII on CSS in UTUC

**Figure 3:**
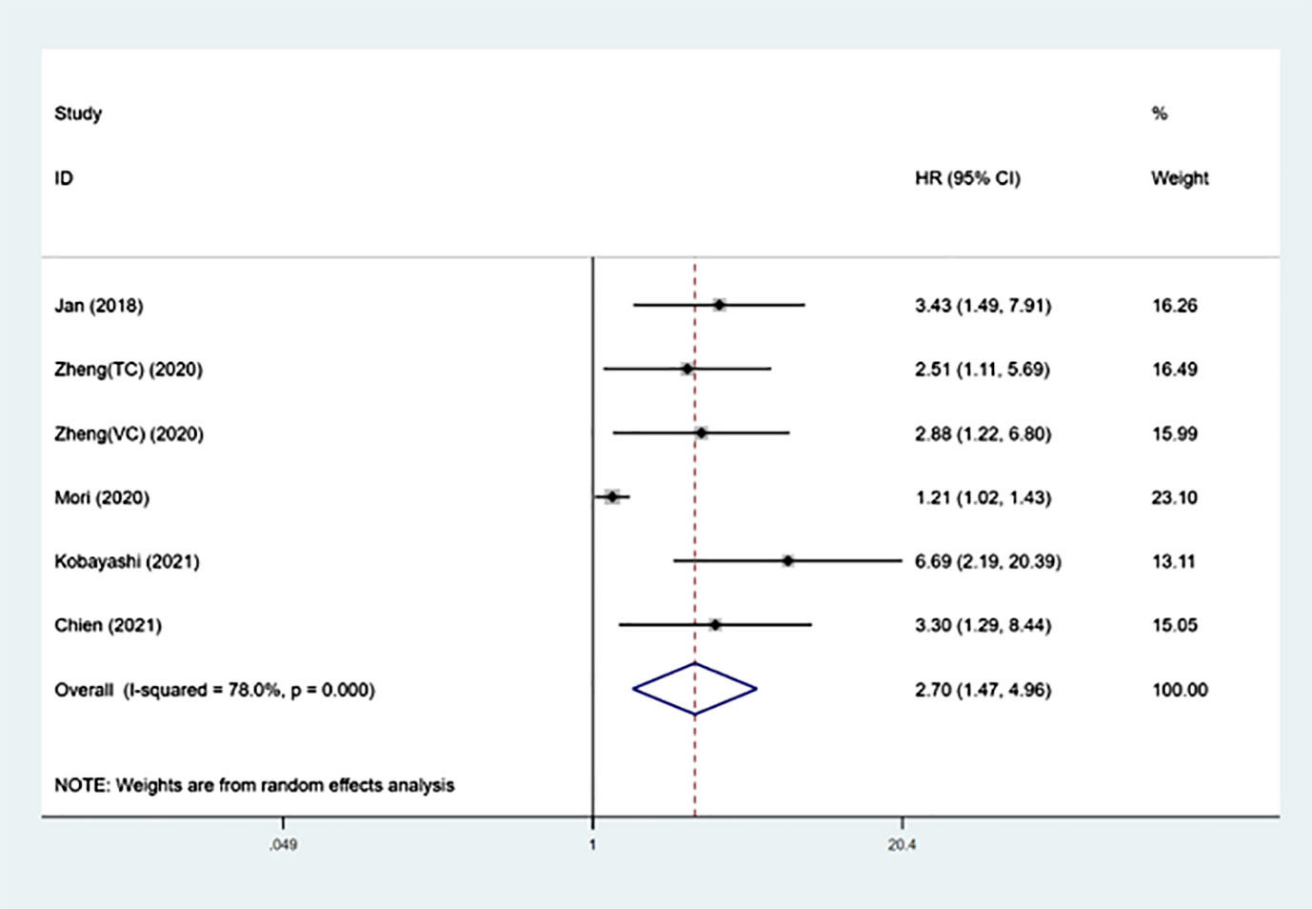
Forest plot of the association between SII and CSS in patients with UTUC.

The prognostic importance of SII for CSS was evaluated by six groups involving 3801 patients (13–17), and the overall findings indicated that increased SII levels were linked to worse CSS outcomes (HR =2.70, 95%CI 1.47-4.96, P=0.001). Despite the notable heterogeneity (I²= 78.0%, p<0.001), we continued to utilize the random-effects model for the combination process (**Figure 3** and **Table 3**). The findings in **Table 3** demonstrate that increased SII was significantly linked to worse CSS in subgroups with sample sizes less than 300 (p<0.001), cut-off values greater than 550 (p<0.001), and median follow-up times exceeding 40 months.

### The predictive importance of SII on RFS in UTUC

**Figure 4:**
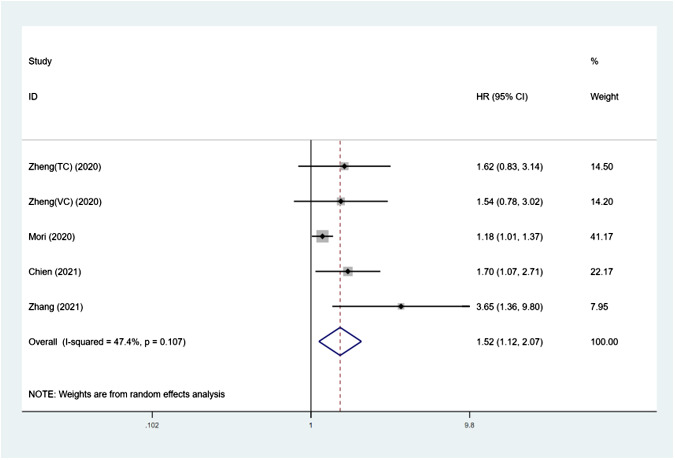
Forest plot of the association between SII and RFS in patients with UTUC.

**Figure 4** shows how the SII affects RFS in UTUC patients, with a total of 5 groups comprising 3384 patients. Using a random-effects model, a combined hazard ratio of 1.52 was computed, with a 95% confidence interval ranging from 1.12 to 2.07 and a significance level of 0.007. This suggests that higher SII levels were linked to worse recurrence-free survival rates (I²= 47.4%, p=0.107) (**Figure 4** and **Table 3**). Further subgroup analysis revealed that when sample size<300 (p=0.008), and median follow-up time > 40 months (p=0.011), SII increase was correlated with poor RFS.

### Correlation of SII with clinicopathological characteristics in UTUC

**Figure 5:**
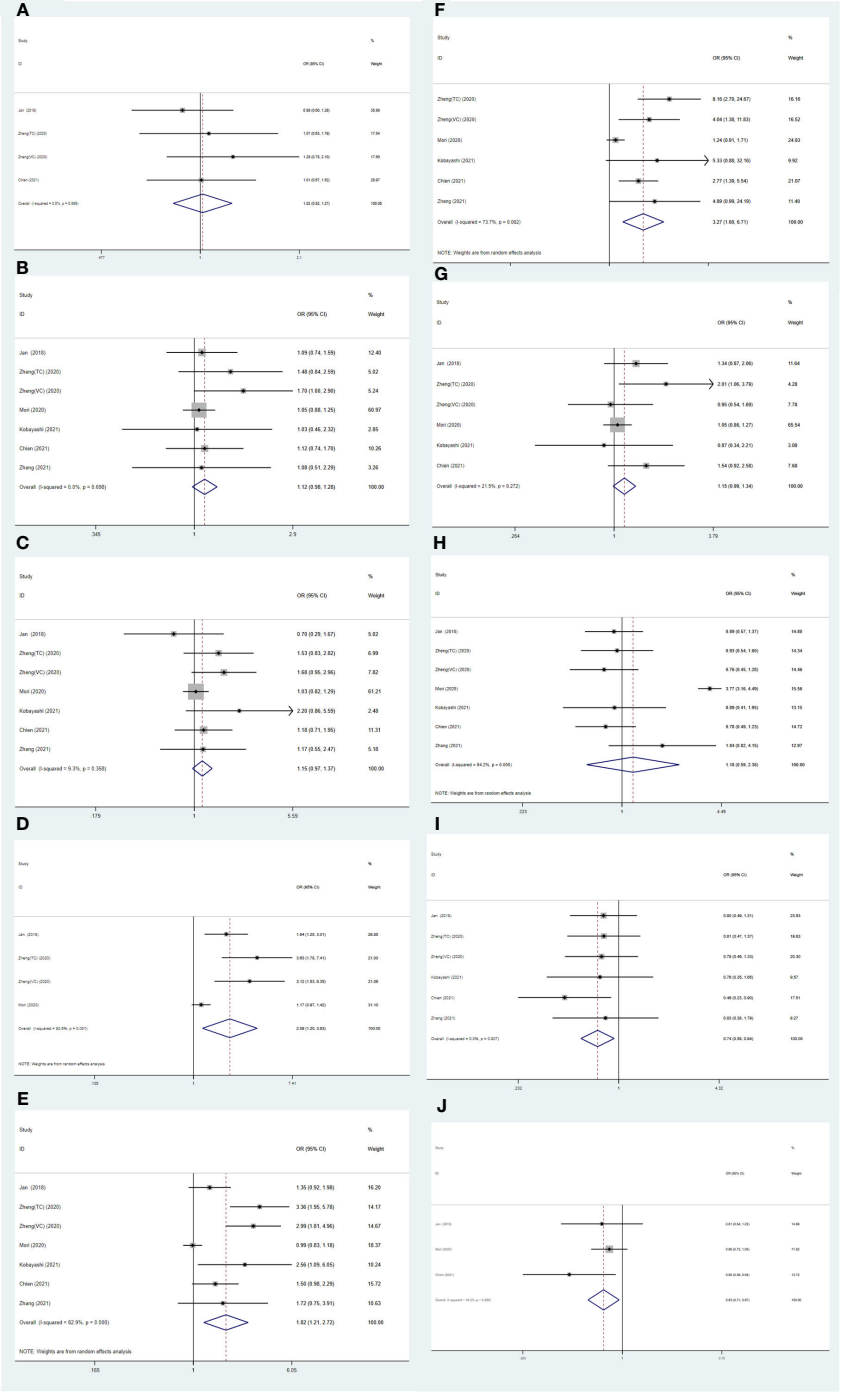
Forest plots of the association between SII and clinicopathological characteristics in UTUC: **(A)** Age (old *vs*. young); **(B)** Gender (male *vs*. female); **(C)** Histological grade (high *vs*. low); **(D)** LVI (present *vs*. absent); **(E)** pT stage (pT ≥ 3 *vs*. < 3); **(F)** pN stage (N+ *vs*. N0); **(G)** Multifocality (present *vs*. absent); **(H)** Tumor location (ureter *vs*. pelvis); **(I)** Hydronephrosis (present *vs*. absent); **(J)** Bladder cancer history (present *vs*. absent).

The findings of additional analysis on the correlation between SII and typical clinicopathological characteristics are displayed in **Table 4** and **Figure 5**. Indicators included in the study are age (old *vs* young), gender (male *vs* female), histological grade (high *vs* low), LVI (present *vs* absent), pT stage (pT≥3 *vs* < 3), pN stage (N+ *vs* N0), multifocality (present *vs* absence), tumor location (ureter *vs* pelvis), hydronephrosis (present *vs* absent), and bladder cancer history (present *vs* absent). The results showed that higher SII may be related to LVI (present *vs* absent) (OR=0.83, 95% CI=0.71-0.97, p=0.018), pT stage (pT≥3 *vs* < 3) (OR=1.82, 95% CI=1.21-2.72, p=0.004), pN stage (N+ *vs* N0) (OR=3.27, 95% CI=1.60-6.71, p=0.001). Hydronephrosis and a history of bladder cancer are viewed as protective factors in cases of Higher SII, with odds ratios of 0.74 (95% CI=0.58-0.94, p=0.013) and 0.83 (95% CI=0.71-0.97, p=0.018) respectively (**Table 4** and **Figure 5**).

**Table 4:** Correlations between SII and clinicopathological characteristics in UTUC.

No.	Characteristics	Cohorts (n)	Patients (n)	Effects model	OR (95% CI)	p-Value	Heterogeneity	
							I² (%)	p-Value
1	Age (old vs. young)	4	1325	Fixed	1.02 (0.82, 1.27)	0.876	0.00%	0.696
2	Gender (male vs. female)	7	3911	Fixed	1.12 (0.98, 1.28)	0.101	0.00%	0.698
3	Histological grade (high vs. low)	7	3911	Fixed	1.15 (0.97, 1.37)	0.108	9.30%	0.358
4	LVI (present vs. absent)	4	3322	Random	2.09 (1.20, 3.63)	0.009	82.60%	0.001
5	pT stage (pT≥3 vs. <3)	7	3744	Random	1.82 (1.21, 2.72)	0.004	82.90%	0.000
6	pN stage(N+ vs. N0)	6	1586	Random	3.27 (1.60, 6.71)	0.001	73.70%	0.002
7	Multifocality (present vs. absent)	6	3801	Fixed	1.15 (0.99, 1.34)	0.076	21.50%	0.272
8	Tumor location (ureter vs. pelvis)	7	3703	Random	1.18 (0.59, 2.38)	0.642	94.20%	0.000
9	Hydronephrosis (present vs. absent)	6	1538	Fixed	0.74 (0.58, 0.94)	0.013	0.00%	0.807
10	Bladder cancer history (present vs. absent)	3	3173	Fixed	0.83 (0.71, 0.97)	0.018	19.30%	0.290

SII, systemic immune-inflammation index; UTUC, upper tract urothelial carcinoma; OR, odds ratio; CI, confidence interval; LVI, lymphovascular invasion.

### Publication bias

**Supplementary Figure S1** displays the utilization of Begg’s tests in assessing the publication bias within the literature that was included. The findings indicated the absence of any apparent bias in publication for OS, CSS, and RFS with p-values greater than 0.05 according to Begg’s test results (OS p=0.221, CSS p=0.060, RFS p=0.462).

### Sensitivity analysis

The reliability of the combined HRs was assessed by conducting a sensitivity analysis using the leave-one-out test on the three outcome measures of OS, CSS, and RFS. The findings indicated that the conclusion was not influenced by any individual study, demonstrating that the collective impact of this meta-analysis was consistent and trustworthy (**Supplementary Figure S2**).

## Discussion

Carcinogenesis is increasingly regarded as a function of the interaction between the tumor and the surrounding tumor microenvironment (TME) (19, 20). Increasing evidence suggests that the development of a tumor frequently signifies an imbalance in the body’s inflammatory immune response, with inflammation being intricately linked to the tumor. The presence of immune cells is crucial for speeding up the growth of tumors, the formation of new blood vessels, and the spread of cancer to other parts of the body through the stimulation of different cytokines. There has been emerging evidence that neutrophils are involved in cancer-related inflammation. Reactive oxygen species (ROS) and reactive nitrogen species (RNS) generated by neutrophil toxicity can lead to DNA damage and genetic instability (21), while neutrophils can further advance tumor growth through enzyme release (22–24). Much literature has shown that platelets promote tumor progression and metastasis (25–27). By identifying tumor-associated antigens, T lymphocytes that infiltrate tumors can initiate immune responses against the tumor (28). Research has demonstrated that tumors with T cell inflammation are more responsive to immunotherapy (29).

The SII provides a more comprehensive measure of inflammation in the body than the previous indexes like PLR, NLR, and LMR. The body’s red blood cell, platelet, and lymphocyte levels are included in it. In the research on hepatocellular carcinoma, SII has been identified as a valuable prognostic marker for the first time (30). Subsequently, it has been verified that it has prognostic value in various tumors, and it is a novel marker with the advantages of simplicity and low price. Numerous past meta-analyses have indicated a correlation between SII and the outcome of cancerous growths, including tumors in the digestive system, urogenital system, lung, pancreas, breasts, and more (31–36). The initial meta-analysis of SII in individuals with UTUC examines its predictive value by pooling information from six studies involving 3911 participants. Despite the limited number of studies included, it is considered adequate considering the rare occurrence of UTUC. Furthermore, all the specimens analyzed in this research consisted of over 100 instances in order to minimize any potential disruptions. Our analysis systematically assessed the potential usefulness of SII in predicting clinicopathological characteristics and prognosis in patients with urinary cancer undergoing treatment with RUN. Our statistical analysis indicated that elevated levels of SII before surgery were more reliable indicators of worse survival results, such as reduced OS, CSS, and RFS, along with negative pathological characteristics like LVI (present *vs* absent), pT stage (pT≥3 *vs* < 3), and pN stage (N+ *vs* N0). Previous articles of the same type have reached similar conclusions (37).

According to the 2020 EAU guidelines, patients with UTUC should be divided into low-risk and high-risk groups according to whether the tumor is solitary, tumor size, tumor pathological grade, and whether there is metastasis on preoperative examination (2). In cases of high-risk UTUC, RNU with bladder excision remains the standard treatment (38). There are still some controversial problems in the clinical diagnosis and treatment of UTUC. Clinically, we usually perform lymph node dissection (LND) for patients with possible lymph node metastasis suggested by preoperative imaging or suspected lymph node metastasis found during surgery. Due to the lack of solid evidence and prospective randomized controlled studies, LND is still controversial. Some studies have shown that LND improves survival, even in patients with clinicopathological negative lymph nodes (39, 40). Some relevant studies have proved that ureteroscopic biopsy before radical nephroureterectomy may increase the risk of postoperative bladder recurrence (41–43). Due to this, computed tomography urography (CTU) has become a first-line examination method to diagnose UTUC, due to its high sensitivity and specificity (44, 45). However, for some atypical advanced UTUC, accurate preoperative diagnosis and clinical staging still face challenges.

Preoperative neoadjuvant chemotherapy and postoperative adjuvant therapy are hot topics in this field. Adjuvant chemotherapy (AC) is chemotherapeutic therapy commonly used to reduce tumor recurrence or metastasis after surgery in patients with malignant tumors. Neoadjuvant chemotherapy (NAC) is systemic chemotherapy before local treatment, such as surgery or radiation. For high-risk upper tract urothelial carcinoma, a growing body of data shows that patients receiving neoadjuvant chemotherapy (NAC) prior to RNU may benefit more (46, 47). A study based on National Cancer Database (NCDB) data from the United States shows that preoperative determination of the depth of a primary upper urinary tract tumor remains to be addressed and is a key factor in determining whether to implement NAC in patients with high-grade UTUC (48). Based on a meta-analysis, NAC was associated with statistically significant OS and CSS in all patients and in patients with locally advanced UTUC. AC was associated with improved metastasis-free survival and CSS patients with locally advanced UTUC. In contrast, the association between AC and OS was significant only patients with locally advanced UTUC (49).

In particular, this study further examined the relationship between SII and clinicopathological characteristics, and found that SII was associated with LVI (present *vs* absent), pT stage (pT ≥ 3 *vs*<3) and pN stage (N+ *vs* N0), indicating the potential value of SII in determining preoperative clinical staging and risk stratification. SII can be included in the prognostic model to increase the accuracy and provide more accurate clinical evidence support for NAC and AC treatment. We advocate that SII can be used as a clinical decision aid until a patient’s treatment is determined. Asian patients had more severe and graded diseases than other ethnic groups (49, 50). However, subgroup analysis in this study showed that race was not yet an independent predictor of survival.

This article still has the following limitations. First, this study included a small number of articles, only 7 cohorts of 6 studies, including 3911 patients. Second, among the six included studies, only one was from Europe and the United States, while the remaining five were from Asia, including Japan, China and Taiwan. Additionally, all included studies were retrospective. The above factors suggest that there may have been publication bias in this meta-analysis. We look forward to further improving the meta-analysis in more large-scale clinical studies.

## Conclusion

According to a comprehensive analysis of all included articles, higher preoperative SII was independently associated with poorer survival outcomes and pathological changes. To some extent, SII can be used as a convenient, cheap and reliable prognostic marker for UTUC patients.

## Data availability statement

The original contributions presented in the study are included in the article/**Supplementary Material**. Further inquiries can be directed to the corresponding authors.

## Author contributions

ZY: Writing – original draft, Writing – review & editing. ZX: Writing – original draft, Writing – review & editing. JM: Writing – original draft, Writing – review & editing. PD: Funding acquisition, Project administration, Resources, Writing – review & editing. SW: Writing – review & editing. JL: Validation, Investigation, Writing – review & editing. YC: Writing – review & editing. YY: Conceptualization, Resources, Writing – review & editing.
