# Supplementary material for: Prognostic and clinicopathological significance of systemic immune-inflammation index in upper tract urothelial carcinoma: a meta-analysis of 3911 patients
Source: Front Oncol. 2024 Jun 14;14:1342996. doi: 10.3389/fonc.2024.1342996 (PMC11211359; doi:10.3389/fonc.2024.1342996)

**Figure S1.** Detection of publication bias in meta-analyses of (**A**) overall survival, (**B**) cancer-specific survival, and (**C**) recurrence-free survival

A：
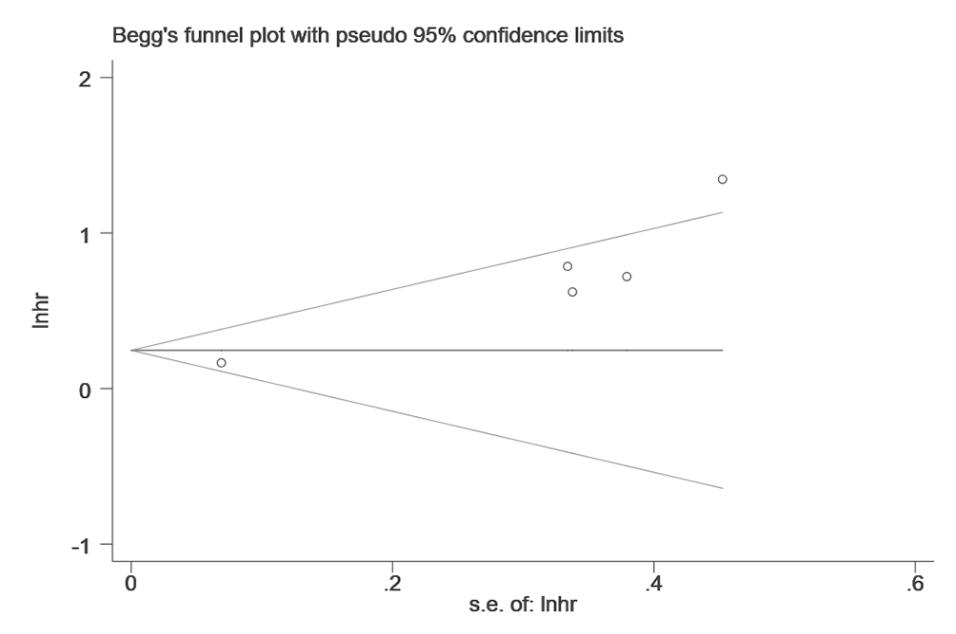


B：
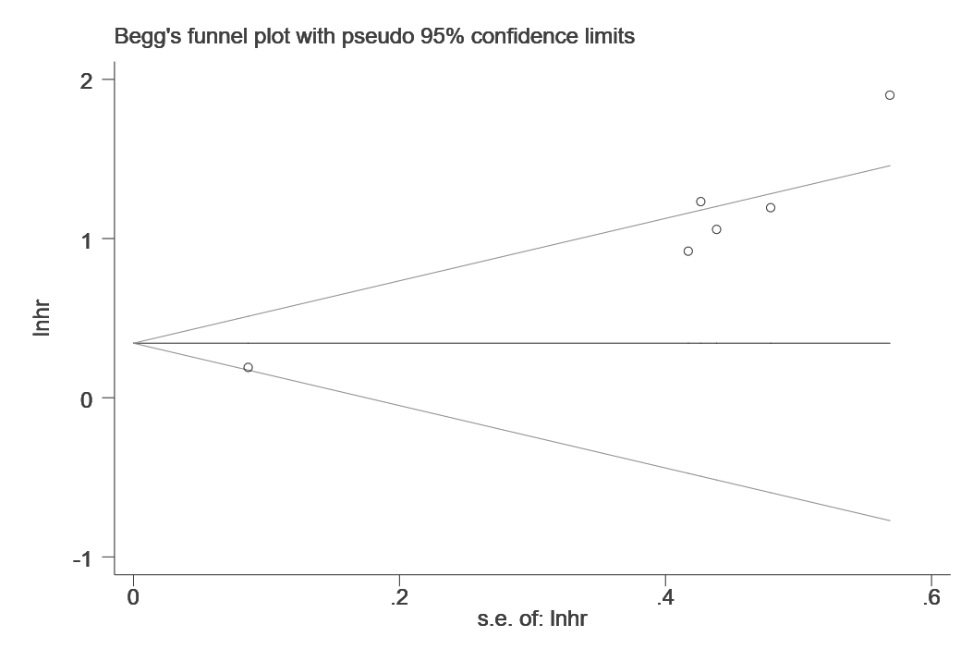


C：
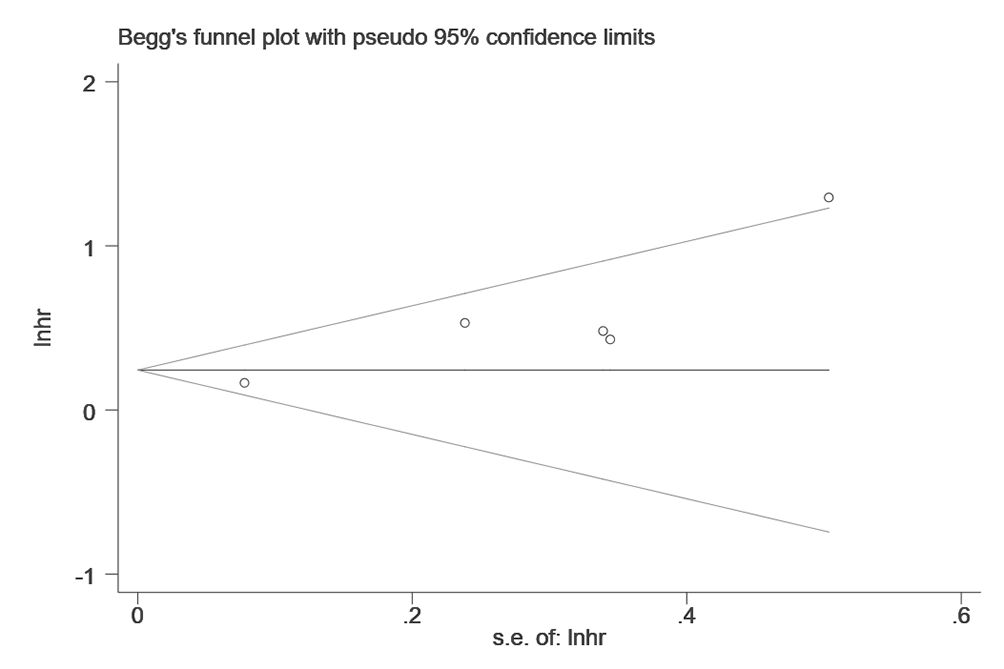


**Figure S2.** Sensitivity analysis of the prognostic impact of systemic immune-inflammation index on (**A**) OS (**B**) CSS, and (**C**) RFS in bladder cancer.

A：
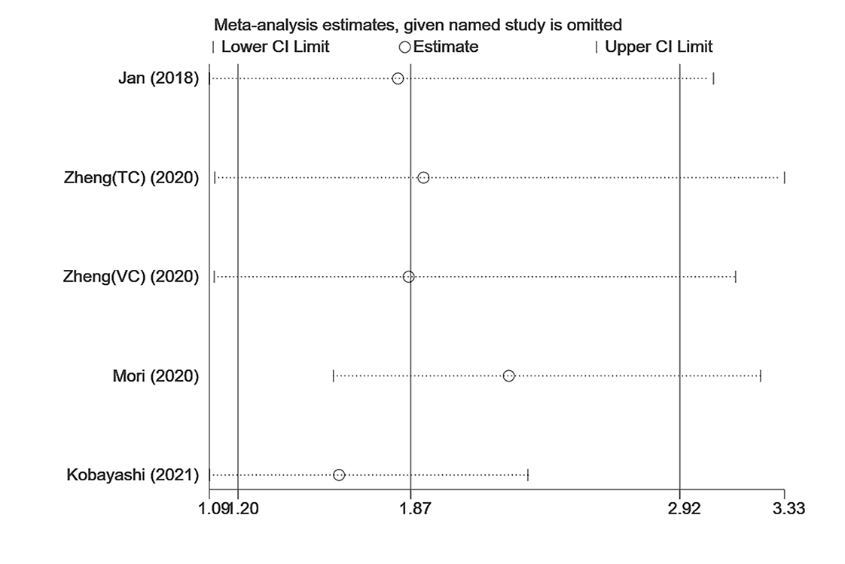


B：
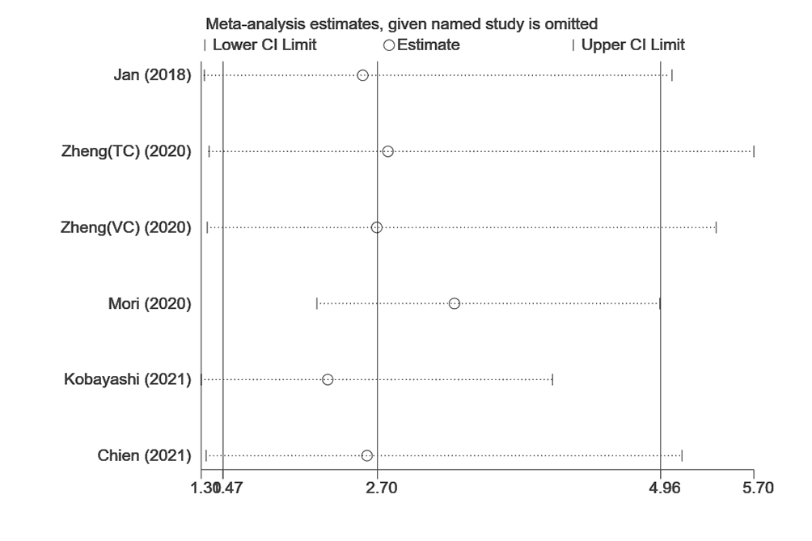


C：
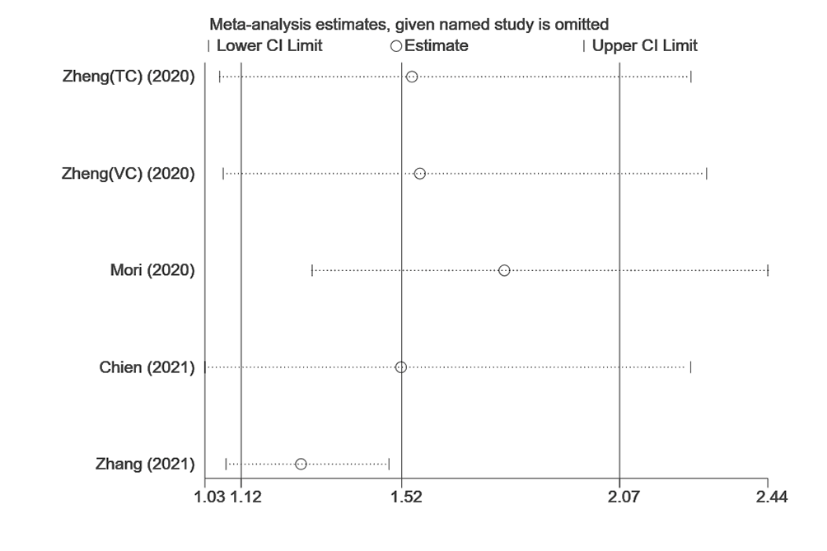

Supplement: Supplementary file 1 [file DataSheet_1.docx]
